# Confronting the loss of trophic support

**DOI:** 10.3389/fnmol.2023.1179209

**Published:** 2023-06-23

**Authors:** Hui-Lan Hu, Latika Khatri, Marilyn Santacruz, Emily Church, Christopher Moore, Tony T. Huang, Moses V. Chao

**Affiliations:** ^1^Department of Biochemistry and Molecular Pharmacology, New York University Langone School of Medicine, New York, NY, United States; ^2^Skirball Institute for Biomolecular Medicine, Neuroscience Institute, New York University Langone Medical Center, New York, NY, United States; ^3^Department of Neuroscience, Pomona College, Claremont, CA, United States; ^4^Department of Cell Biology, New York Langone Medical Center, New York, NY, United States; ^5^Department of Psychiatry, New York Langone Medical Center, New York, NY, United States; ^6^Department of Neuroscience and Physiology, New York Langone Medical Center, New York, NY, United States

**Keywords:** neurotrophins, nerve growth factor, brain derived neurotrophic factor, sympathetic neurons, hippocampal neurons, longevity, growth factors, receptors

## Abstract

Classic experiments with peripheral sympathetic neurons established an absolute dependence upon NGF for survival. A forgotten problem is how these neurons become resistant to deprivation of trophic factors. The question is whether and how neurons can survive in the absence of trophic support. However, the mechanism is not understood how neurons switch their phenotype to lose their dependence on trophic factors, such as NGF and BDNF. Here, we approach the problem by considering the requirements for trophic support of peripheral sympathetic neurons and hippocampal neurons from the central nervous system. We developed cellular assays to assess trophic factor dependency for sympathetic and hippocampal neurons and identified factors that rescue neurons in the absence of trophic support. They include enhanced expression of a subunit of the NGF receptor (Neurotrophin Receptor Homolog, NRH) in sympathetic neurons and an increase of the expression of the glucocorticoid receptor in hippocampal neurons. The results are significant since levels and activity of trophic factors are responsible for many neuropsychiatric conditions. Resistance of neurons to trophic factor deprivation may be relevant to the underlying basis of longevity, as well as an important element in preventing neurodegeneration.

## Introduction

Neurons are post-mitotic cells with a limited regenerative capability; however, each cell has the capacity of survival for a lifetime. How neurons persist during a life span raises the question of how neurons can survive when there is a loss of trophic support. It is well established that expression of trophic factors increases the resistance of neurons to neurodegeneration and aging (Barde, 1989). During development, dependence on trophic support is based upon the excess production of vertebrate neurons over the number of targets. A pruning of neurons occurs during early development to match the innervation of targets by peripheral neurons. Elimination of excess neurons through programmed cell death occurs from competition of limiting amounts of trophic factors and growth factors present at the target (Jacobson et al., 1997).

A classic developmental example is sympathetic neurons, which are critically dependent on NGF for survival. Sympathetic neurons isolated from the superior cervical ganglia (SCG) of embryonic rodents undergo apoptotic cell death in response to NGF deprivation (Martin et al., 1988; Deckwerth and Johnson, 1993). As the cells mature *in vitro* and *in vivo*, however, these neurons develop a resistance to trophic factor deprivation and become much less acutely dependent on NGF for survival (Lazarus et al., 1976; Goedert et al., 1978). Little is understood about how neurons become independent of trophic support.

Sympathetic neurons uniformly express p75 and TrkA NGF receptors (Murray et al., 2004), both of which participate in neuronal survival through high affinity picomolar NGF binding (Hempstead et al., 1991). TrkA receptors have been detected in both young and sympathetic mature neurons, with a pattern of phosphorylation that persists in mature cells (Tsui-Pierchala and Ginty, 1999). TrkA receptors can be downregulated by ubiquitination, which abrogates its survival function (Arevalo et al., 2006). In addition, the p75 neurotrophin receptor is colocalized with TrkA in sympathetic neurons and promotes high affinity binding with TrkA (Hempstead et al., 1991). Hence these two receptors play a central role in the survival decisions of sympathetic neurons.

How neurons avoid cell death after a loss of trophic support is a fundamental problem in biology. It is well established sympathetic neurons undergo rapid programmed cell death in response to NGF deprivation that requires the involvement of Bcl-2 members and caspase activation (Deckwerth and Johnson, 1993). However, cultures maintained for over 3–4 weeks develop a resistance to NGF deprivation and become much less dependent upon NGF for survival (Chun and Patterson, 1977; Easton et al., 1997). After withdrawal of NGF, mature neurons do not experience a loss of survival, but they do undergo biochemical and transcriptional changes elicited by NGF deprivation. A switch in trophic factor requirements also occurs *in vivo* after deprivation of trophic factors (Angeletti et al., 1971; Lazarus et al., 1976; Goedert et al., 1978). The basis of this switch in dependence upon trophic factors is not known, even though this event was first reported over 50 years ago.

An overlooked and fundamental question is what accounts for the ability of mature sympathetic neurons to avoid cell death when deprived of NGF. Despite considerable research on the mechanism of action of growth factors, the question about how neurons switch to become independent of trophic factors has not been fully addressed. This outcome can be observed in long term cultures of sympathetic neurons. In contrast to cell death that occurs in young neurons after NGF withdrawal, negligible cell loss occurs in NGF-deprived mature cultures (Lazarus et al., 1976; Chun and Patterson, 1977), either by removal of NGF or treatment with anti-NGF antibodies. An increased resistance to trophic factor loss has been described for a number of neuronal populations and represents an important protective mechanism for maintenance of the nervous system by growth factors (Rich et al., 1987; Snider, 1994; Kuzis et al., 1999).

We hypothesize that changes in neuronal gene expression represent elements that account for the resistance to trophic factor deprivation. To this end, we developed cellular assays to assess trophic factor independence for sympathetic and also hippocampal neurons, as a representative of the CNS. The ability of neurons to resist apoptosis in the absence of trophic factors may be a key event in avoiding cell death. These changes may represent a potential protective mechanism for maintenance of the nervous system and may also explain how neuronal populations become vulnerable to neurodegeneration. Here, we describe the identification of several factors that can rescue neurons from an absence of trophic support. They include expression of a specific NGF receptor isoform and the glucocorticoid receptor, which have been implicated in cell survival and transcriptional regulation. The ability to reverse the dependency upon trophic support with different signals suggests that multiple factors may contribute ability to withstand a loss of trophic support.

## Results

**Figure 1:**
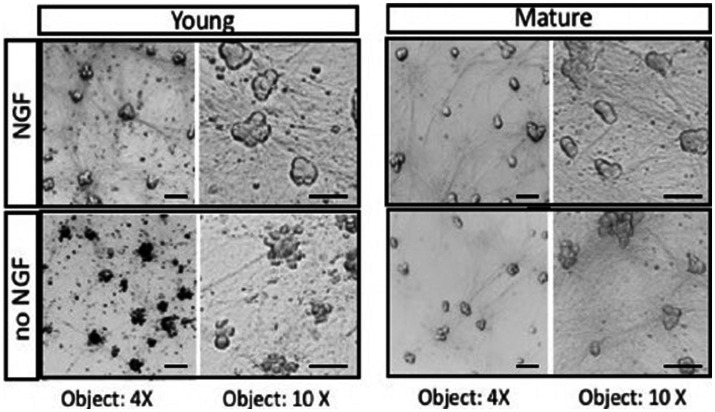
Neurons isolated from E21 embryonic SCG undergo apoptosis in response to NGF deprivation. Young neurons were cultured for 5 days in the presence of 50 ng/mL NGF. After removal of NGF (no NGF), neurons underwent rapid cell death. Mature neurons maintained for over 2 weeks *in vitro* with NGF become resistant to NGF deprivation (no NGF). At the light microscope level, mature sympathetic neurons displayed a decrease in cell diameter and cell volume. Similar morphological results were obtained with adult monkeys treated with anti-NGF antibodies (Belanger et al., 2017). Scale bar = 50um.

The fundamental question of how mature neurons are capable of avoiding cell death when deprived of trophic support can be studied *in vitro* using cultures of embryonic sympathetic neurons (Birren et al., 1993). Sympathetic neurons provide several advantages including the ability to be cultured as a nearly pure population of neurons. They are a useful model system because of their homogeneity and experimental accessibility. The experiment shown in Figure 1 was carried out to demonstrate that deprivation of sympathetic neurons can be repeated by removal of NGF, as was reported many years ago. Removing trophic support from sympathetic neurons reproducibly leads to apoptotic cell death within 2 days. Sympathetic neurons grown for a short period of time (< 5 days, Young) undergo rapid cell death in the absence of NGF, whereas cultures maintained for over 3–4 weeks (Mature) do not experience a loss of viability (Figure 1). Over time, sympathetic neurons become independent of NGF deprivation (Easton et al., 1997).

The cell culture results indicate that mature neurons become less sensitive to NGF withdrawal as they grow older. As the cells mature *in vitro* and *in vivo*, however, they develop a resistance to NGF deprivation with time. Sympathetic neurons maintained in culture for 3–4 weeks become largely resistant to NGF deprivation. The basis of the switch in trophic factor dependence upon trophic factors is not known. One possibility is that maturation of neurons gives rise to a greater resistance in their capacity to undergo cell death (Hollville et al., 2019). The machinery responsible for apoptosis include caspases, Bcl-2 family members, and mitochondrial function (Deckwerth and Johnson, 1993; Johnson et al., 1996), are likely to be modulated to give trophic factor independence in neurons.

What changes in peripheral mature neurons account for independence to NGF? We will focus on sympathetic neurons to address this question. Peripheral sensory and sympathetic ganglia have served as useful model systems for studying these issues because of their relative simplicity and experimental accessibility. We established long-term cultures of mature sympathetic neurons, which reproducibly lose their dependence upon NGF for survival. The goal is to identify the changes that occur during and after their reliance upon NGF.

### Other trophic factors

A plausible explanation why mature neurons survive for an extended time in the absence of NGF is that other trophic factors replace the loss of NGF. Other factors can arise from non-neuronal cells in culture. We have developed an assay to identify factors that rescue the consequences of deprivation of NGF trophic support.

**Figure 2:**
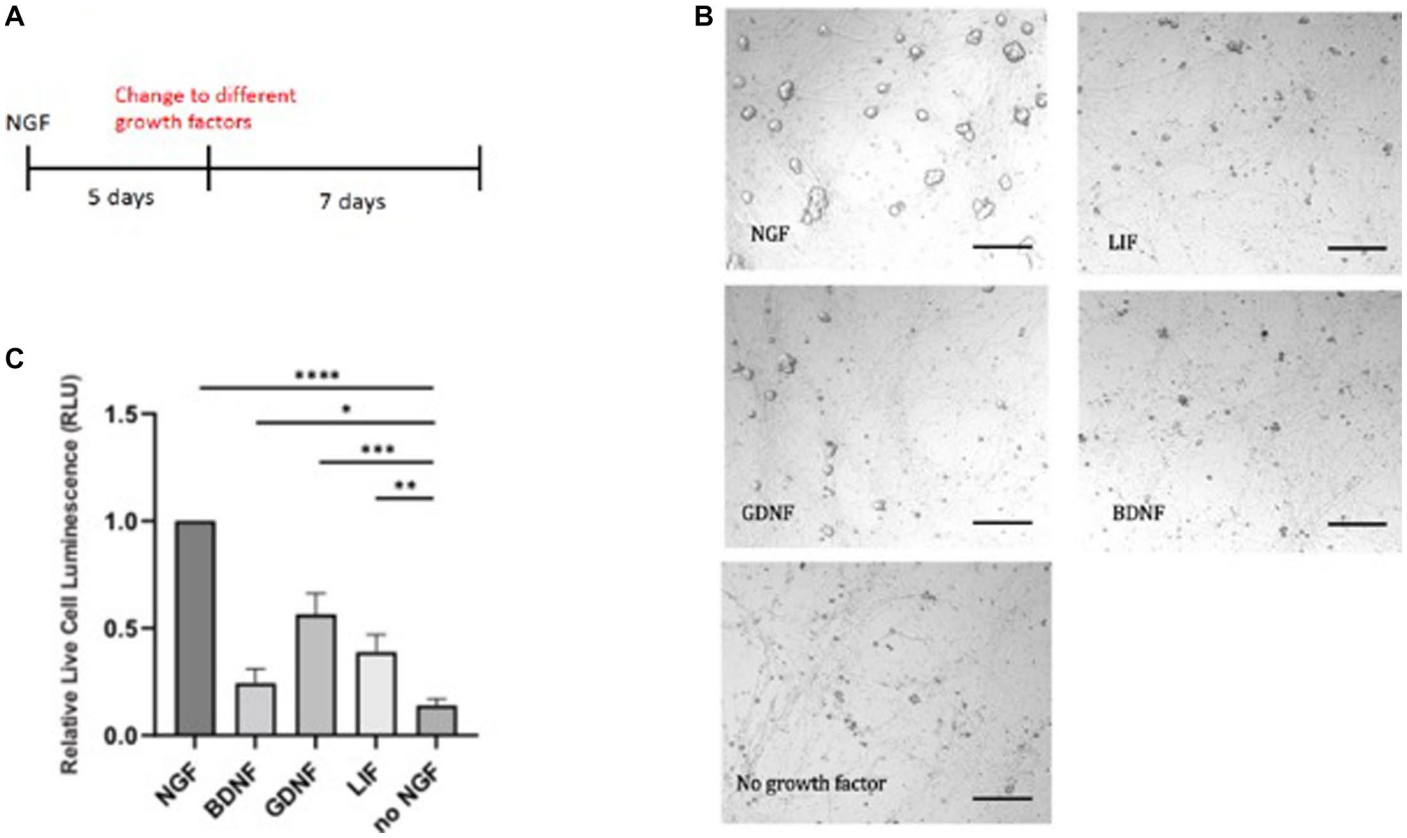
E20 sympathetic neurons were first exposed to NGF for 5 days and then switched to either GDNF, LIF, or BDNF **(A)**. Limited survival was seen in the presence of GDNF, as visualized by the necrotic cells **(B)**. Cell survival was quantitated by a luminescent cytotoxicity assay that measures the number of dead cells in a population. **(C)** Quantitation was done with Prism using one-way ANOVA. Growth factor concentrations used: NGF 50 ng/mL, BDNF 50 ng/mL, GDNF 10 μg/mL, and LIF 25 μg/mL. Scale bar = 200 μm. *p value 0.0132, **p value 0.0013, ***p value 0.0002, and ****p value < 0.0001.

We chose several prominent factors, which have been implicated in sympathetic cell survival. We used 50 ng/mL NGF which is derived from dose response studies carried out on sympathetic neurons (Lockhart et al., 1997). The optimal concentration for SCG primary cultures was determined for their physiological effects on survival and synaptic transmission. These effects were traced to the TrkA receptor. Sympathetic neurons are dependent upon trophic support by NGF and also glial-derived neurotrophic factor, GDNF. GDNF promotes the survival of sympathetic, parasympathetic, and sensory neurons (Buj-Bello et al., 1995). GDNF is a ligand that binds to the RET tyrosine kinase receptor and an auxiliary α-GRF co-receptor protein. We therefore tested different growth factors instead of NGF (Figure 2A). Because of the absence of the TrkB receptor in sympathetic neurons, addition of BDNF, the close relative of NGF, did not give a rescue in the absence of NGF. Without addition of growth factors, there was no rescue seen in the absence of NGF. In this assay, GDNF is capable of partial support in the absence of NGF (Figures 2B,C).

To extend these results, we tested the ability of leukemia inhibitory factor, LIF, which exhibits trophic activity for ciliary, motor, and hippocampal neurons. The LIF cytokine gave nominal support (Figures 2B,C), in contrast to earlier reports of survival of embryonic and postnatal sympathetic neurons (Kotzbauer et al., 1994). These experiments provide evidence that NGF is a superior trophic factor for sympathetic neurons, but suggests alternative networks may be used when these neurons become independent of trophic support.

### Analysis of young vs. mature sympathetic neurons

Following trophic factor withdrawal, sympathetic neurons continue to synthesize protein and RNA. The death of NGF-deprived neurons is strikingly prevented by inhibiting protein or RNA synthesis (Martin et al., 1988). Cycloheximide, puromycin, anisomycin, and actinomycin D all prevent neuronal death subsequent to NGF deprivation as assessed by morphologic and biochemical criteria (Park et al., 1998; Liu et al., 2004). We therefore sought to determine changes that occur during a switch in dependency. The fact that sympathetic neurons synthesize protein and RNA when deprived of trophic factors suggests that NGF and presumably other neurotrophic factors maintain neuronal survival by suppressing an endogenous, active death program.

Responses of neural cells from the environment are mediated by metabolic events. In this regard, mitochondria play a central role in controlling neuroplasticity, neurotransmitter release, and dendritic remodeling (Cheng et al., 2010). Recent evidence suggests that NGF exerts neurotrophic activity by influencing cell metabolism (Sun et al., 2017; Colardo et al., 2022). As mitochondria are ATP generating organelles that are distributed throughout the length of axons and in presynaptic terminals, they participate in fission and fusion events as well as neurite outgrowth.

To assess the changes from immature to mature sympathetic neurons, we initiated RNA-seq experiments on sympathetic neurons grown under early and longer culture conditions, as described in Figure 1. RNA-seq is widely used for measuring gene expression. To date, it has not been applied to this problem. Due to homogeneity of the SCG, we analyzed bulk sympathetic cultures grown for 5 days, followed by removal of NGF for 6 h, defined as immature or young. Parallel cultures were also grown for 3 weeks in the presence of NGF followed by removal of NGF for at least 2 weeks. These cultures are referred to as mature. The SCG was harvested from E18 rat pups, dissociated and plated. The cells were treated with antimitotic agents for 5 days and RNA was subsequently harvested using Trizol.

The RNAseq experiments were carried out with either Young (5 days) or Mature (3–4 weeks) timepoints from the same set of dissected cells. RNA extraction for all samples were carried out at the same time with the same reagents. Three biological replicates for each condition (e.g., 5 days or 3–4 weeks) are collected for analysis.

**Figure 3:**
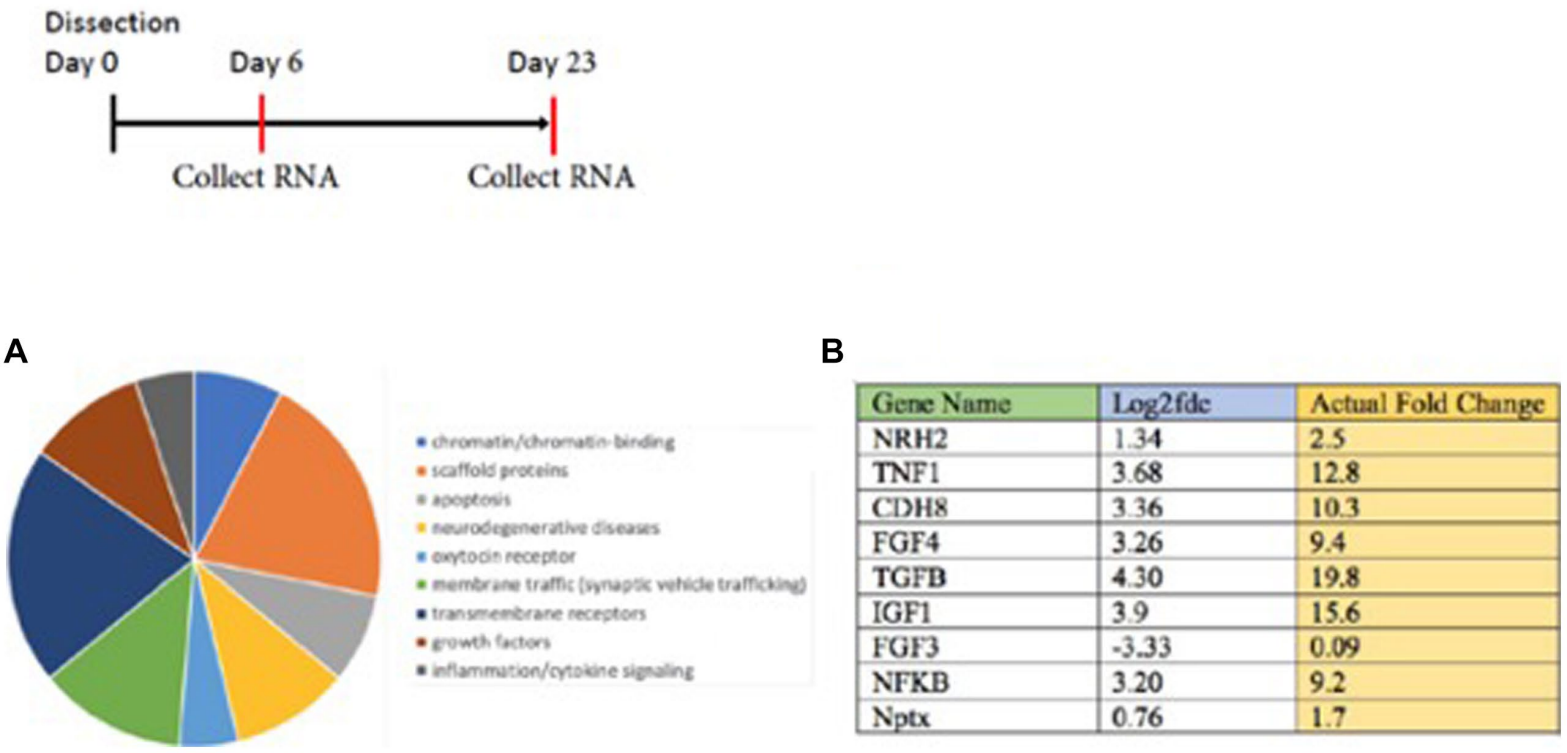
RNA seq analysis is carried out on young vs. mature sympathetic cultures grown for 6 vs. 23 days. **(A)** The results were classified by a pie chart summary of classes of proteins (http://www.pantherdb.org). **(B)** Enhanced levels of these RNAs were observed in sympathetic neurons grown to a mature state. Individual genes are singled out in the Table **(B)**. The analysis indicated the majority of these RNAs showed an increase in expression over younger sympathetic neurons. Ingenuity Pathways Analysis (IPA, NIH) is used as a bioinformatics program to analyze the high-throughput RNA-seq results. The sequencing was carried out by the NYU Genome Center. The sequence reads (fragments/kilobase of transcript) was designated the Fold Change (FC) and converted to the log_2_ fold change in Excel = Log (FC, 2) to get the log_2_ fold change value.

RNA-seq analysis of young vs. mature sympathetic neurons revealed several genes with a higher level of mRNA expression in mature neurons. They include the oxytocin receptor and a number of transmembrane proteins. A pie chart (Figure 3A) summarizes pathways relevant in signaling in sympathetic neurons, along with a schematic of the time course. Prominent increases in gene expression increased in mature sympathetic neurons detected from the RNA-seq analysis are listed in Figure 3B. Comparative RNA analysis indicated the majority of these RNAs showed at least a 2–3-fold increase in expression of mature neurons over young sympathetic neurons.

Interestingly, many of the increases after NGF withdrawal were mRNAs encoding cytokines or growth factors (TNF1, FGF4, TGFβ, and IGF1) or regulatory proteins (CDH8, NFκB, and Nptx). Log ratios are used for the analysis of fold changes from the RNA-seq data. The results are sorted and differential expression results are identified using GFOLD-DifferentialGeneExpression. The log 2-fold change and the actual fold changes for TNF1, FGF4, TGFβ, and IGF1 indicate that an increased synthesis of polypeptide ligands occurred during the prolonged culture of sympathetic neurons. These results also suggest that increases of these proteins may be a response to the withdrawal of NGF from sympathetic neurons.

**Figure 4:**
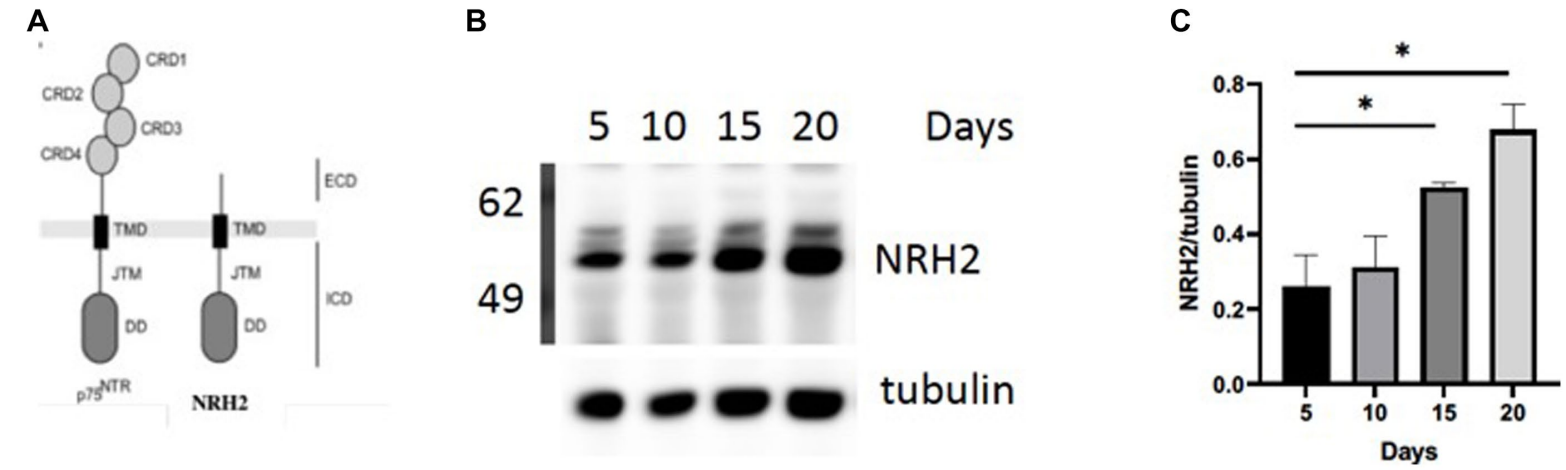
**(A)** NRH2 has a close homology to the p75 receptor. NRH2 contains a similar ICD intracellular domain with the juxtamembrane domain (JMT) and a C-terminal DD (death domain). **(B)** Western blot of matures sympathetic samples after 5–20 days in culture. **(C)** Quantitation of NRH2 levels. Statistical analysis was carried out with Prism using one-way ANOVA. d5 vs d20 *, *p* value 0.0314; d5 vs d15 *, *p* value 0.0471.

In order to validate this RNA-seq approach, we chose to focus on a gene referred to as *Neurotrophin Receptor Homolog-2* (NRH2). The table in Figure 3B indicated a 1–2 fold increase in mRNA for NRH-2 in mature sympathetic neurons. A time course of sympathetic neurons grown for 5–20 days reaffirmed the rise in NRH2 protein expression by western blot analysis (Figure 4B).

### NRH2, proof of principle

There are several reasons for focusing on NRH2. The observed increase in NRH2 protein in the absence of NGF supports the RNA-seq strategy to identify proteins that appear in mature sympathetic neurons. Because of its close structural relationship of NRH2 to the p75 receptor (Figure 4A), we were drawn to the functional significance of an increase of NRH2. Interestingly, previous studies found that co-expression of NRH2 and TrkA led to an enhanced high affinity binding of NGF specifically to TrkA receptors (Murray et al., 2004) and TrkA signaling (Wong et al., 2008). Lacking an extracellular domain (ECD), NRH2 does not bind NGF, but nevertheless is interacting with both TrkA and p75 receptors (Murray et al., 2004; Vilar et al., 2014). Survival of sympathetic neurons requires both TrkA and the p75 receptor, which enhances the sensitivity of these neurons to NGF (Horton et al., 1997). NRH2 has particular relevance to this story, since it contains a consensus death domain (DD) sequence like p75, which has been implicated in both survival and cell death decisions (Hashimoto et al., 2004; Kim and Hempstead, 2009).

**Figure 5:**
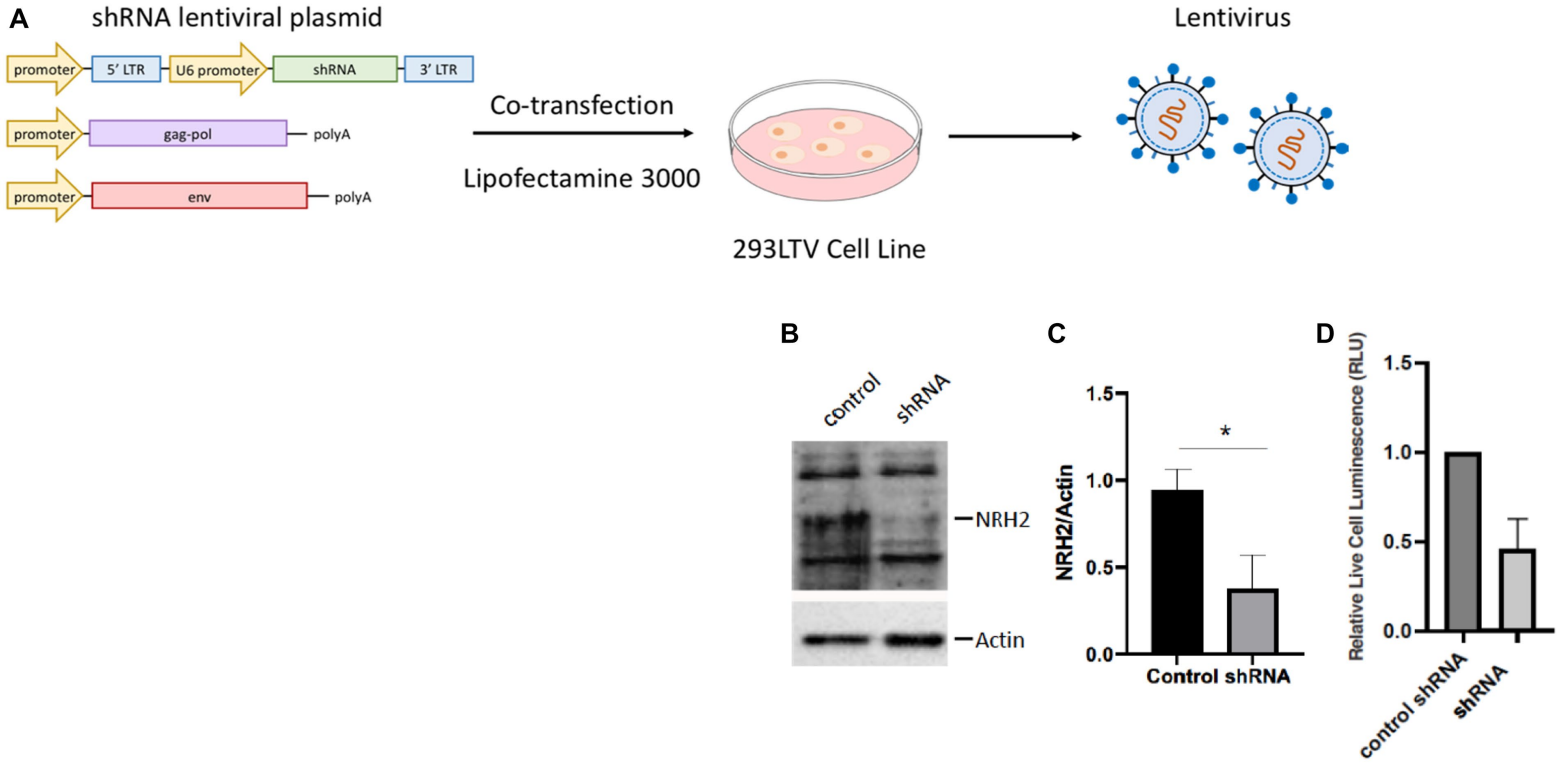
**(A)** A lentivirus cell line (HEK293 LTV) is transiently co-transfected with an NRH2 shRNA lentiviral plasmid, along with envelope and packaging plasmids to generate lentiviral particles. After co-transfection, the virus is collected, stably transduced, and expanded. **(B)** Knockdown of NRH2 in sympathetic neurons by shRNA is verified by western blot, with actin as a control. **(C)** Quantitation indicated a decrease of wild type NRH2 protein with shRNA. Statistics *t*-test *p* = 0.0136 (^*^) **(D)** A cytotoxicity test indicated a decrease in live sympathetic neurons in response to the shRNA for NRH2. A control shRNA did not change the expression of NRH2 or the survival of sympathetic neurons.

To address whether NRH2 levels has an effect upon sympathetic function, we generated lentiviruses using the 293LTV packaging cell line to express shRNA against NRH2. The discovery that NRH2 is participant of trophic receptor signaling and is increased with trophic factor withdrawal suggests the protein may act to influence the survival of sympathetic neurons. To test this hypothesis, we downregulated NRH2 in sympathetic neurons and assessed the viability after shRNA treatment (Figure 5).

Removal of the NRH2 protein by shRNA silencing resulted in a decrease in sympathetic neuron survival. A similar effect was observed when a NGF binding mutant was used on sympathetic neurons (Horton et al., 1997). Since the NGF mutant protein did not bind avidly to the p75 receptor, the mutant NGF interfered with TrkA signaling resulting in cell death. These results suggested the sensitivity of NGF-dependent embryonic sympathetic neurons to NGF, like the effects of NRH2, affected the survival of sympathetic neurons. The ability of NRH2 to increase high affinity NGF binding sites for TrkA (Hempstead et al., 1991; Murray et al., 2004) indicates greater sensitivity can be obtained when NRH2 is elevated after withdrawal of NGF.

We have confirmed that levels of NRH2 represent a switch in trophic support for sympathetic neurons. There are several explanations for the increased resistance of mature neurons to NGF deprivation. Mature neurons may survive for an extended time in the absence of NGF because of other sources of trophic factors (Figure 2). An assumption is that other trophic factor(s) from peripheral targets sustain neurons in maturity. An alternative mechanism is the activity of other pathways, such as endogenous MAP kinase or NFκB activities, which may give rise to survival signals. A loss or change of receptors may also underlie a lack of responsiveness to trophic signals (Birren et al., 1993). Likewise, downstream signaling pathways can be enhanced by a ligand-independent receptor to keep these neurons alive (Mitre et al., 2022). Whether extrinsic or the intrinsic pathways predominant in the switch between dependency and independence from trophic factors needs to be further addressed.

**Figure 6:**
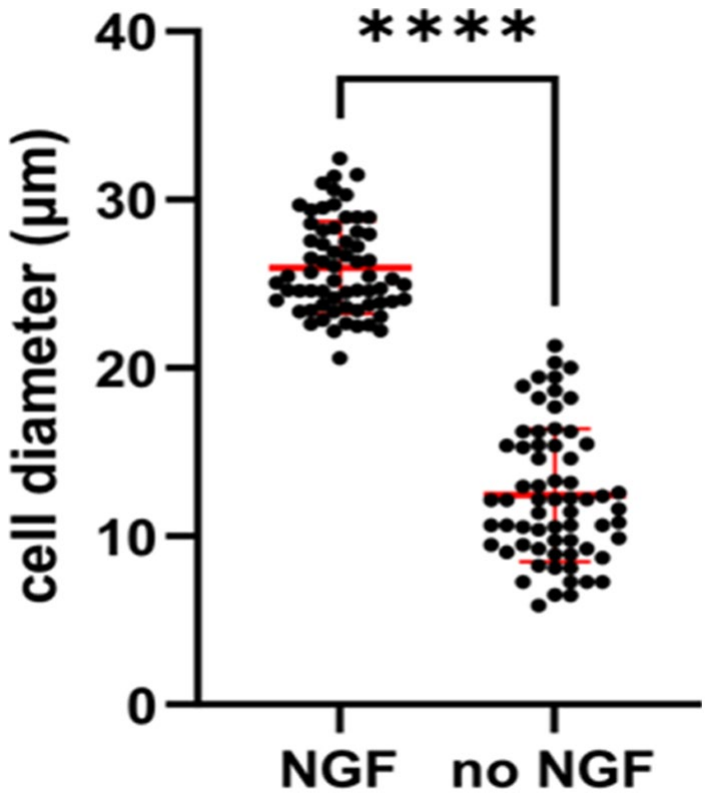
Quantification showing cell diameters of young SCG neurons were significantly smaller in response to NGF deprivation for 2 days. E20 SCG neurons were cultured for 5 days in the presence of 50 ng/mL NGF and then cultured in media with or without NGF (no NGF) Cell diameter were measured by ImageJ and statistics was conducted by GraphPad Prism two-tailed *t*-test *p* < 0.0001. Withdrawal of NGF *in vivo* also resulted in a significant decrease in the soma diameters in the SCG (Belanger et al., 2017).

Independence from trophic factors also give physiological changes in sympathetic neurons. It has been noticed by several groups that mature sympathetic neurons undergo a decrease in soma size in the absence of NGF. An examination of sympathetic neurons deprived of NGF *in vitro* gave rise to a smaller diameter of the soma size (Figure 6). After NGF is withdrawn for 2 days, sympathetic neurons begin to become sick and result in the mitochondrial pathway of apoptosis (Deshmukh et al., 1986). Earlier studies verified the dying SCGs became smaller and display activation of caspase-3 (Kristiansen and Ham, 2014; Figure 1A).

The results shown in Figure 6 are consistent with previous *in vivo* experiments, in which adult sympathetic neurons underwent a decrease in soma size after NGF deprivation of mice, rats and monkeys (Otten et al., 1979; Ruit et al., 1990; Easton et al., 1997; Belanger et al., 2017).

### Trophic support for hippocampal neurons

The switch in responsiveness of sympathetic neurons raises the question whether other neuronal populations might exhibit a similar property. Although PNS neurons have been extensively studied in the context of cell death mechanisms, the vulnerability of individual populations in the CNS is more complex and has not been fully documented, except for hippocampal neurons (Roussarie et al., 2020).

Hippocampal neurons express TrkB receptors and respond to BDNF. BDNF is traditionally ascribed as a main trophic factor for hippocampal neurons. Surprisingly, mice that lack BDNF postnatally displayed negligible effects upon neuronal survival in the mature CNS (Rauskolb et al., 2010). When conditional postnatal BDNF knockout animals were examined, there was little evidence that survival of hippocampal neurons was compromised. The lack of a trophic effect by BDNF implies that hippocampal neurons may be able to withstand the loss of trophic support, in a similar manner as sympathetic neurons.

**Figure 7:**
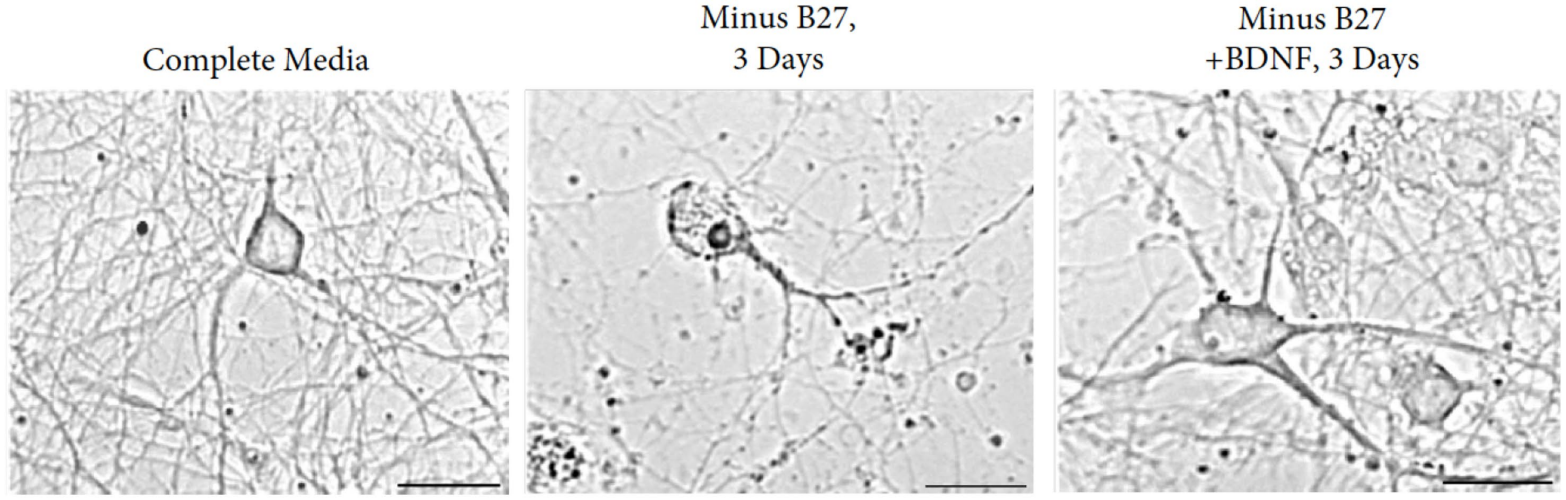
Removal of B27 supplement interferes with the survival of E17 hippocampal neurons. Augmentation with BDNF rescues the detrimental effects of B27-deprived media after 3 days of withdrawal. Young cultures were grown in Neurobasal with B27 media for 3 days. Scale bar = 100 μm.

To define the responses of hippocampal neurons in the absence of trophic support, we resorted to a deprivation protocol with the B-27 supplement. As a defined mixture of reagents, B-27 is composed of 20 components in serum-free media, designed for the survival of embryonic hippocampal neurons. The principal ingredients are insulin, glutathione, the T3 hormone, progesterone, and putrescine (Brewer et al., 1993). Excellent long-term viability occurs after 4 weeks in culture in the presence of B27 media. In addition to the hippocampus, we find embryonic neurons from the cortex; dentate gyrus and striatum are also supported by B-27 media. Cultures of hippocampal neurons were regularly prepared from E18 mouse fetuses and maintained in medium containing B27 supplement (Brewer et al., 1993). A clear dependency exists--when B27 supplement is removed from the medium, hippocampal neurons underwent reproducible cell death within 1 week (Figure 7).

Based on the contrasting effects of B27 supplement and BDNF, we extended the time course from 3 to 28 days to investigate whether independence to BDNF can be obtained in the absence of B27. We found that withdrawal of B27 led to cell death within 48 h, resulting in hippocampal neuron fragmentation with swollen and granular processes. Addition of BDNF to cultures of B27-depleted hippocampal neurons reversed the cell death in the cultures (Figure 7). To exclude external influences, glial contamination is eliminated by 5-fluorouracil.

**Figure 8:**
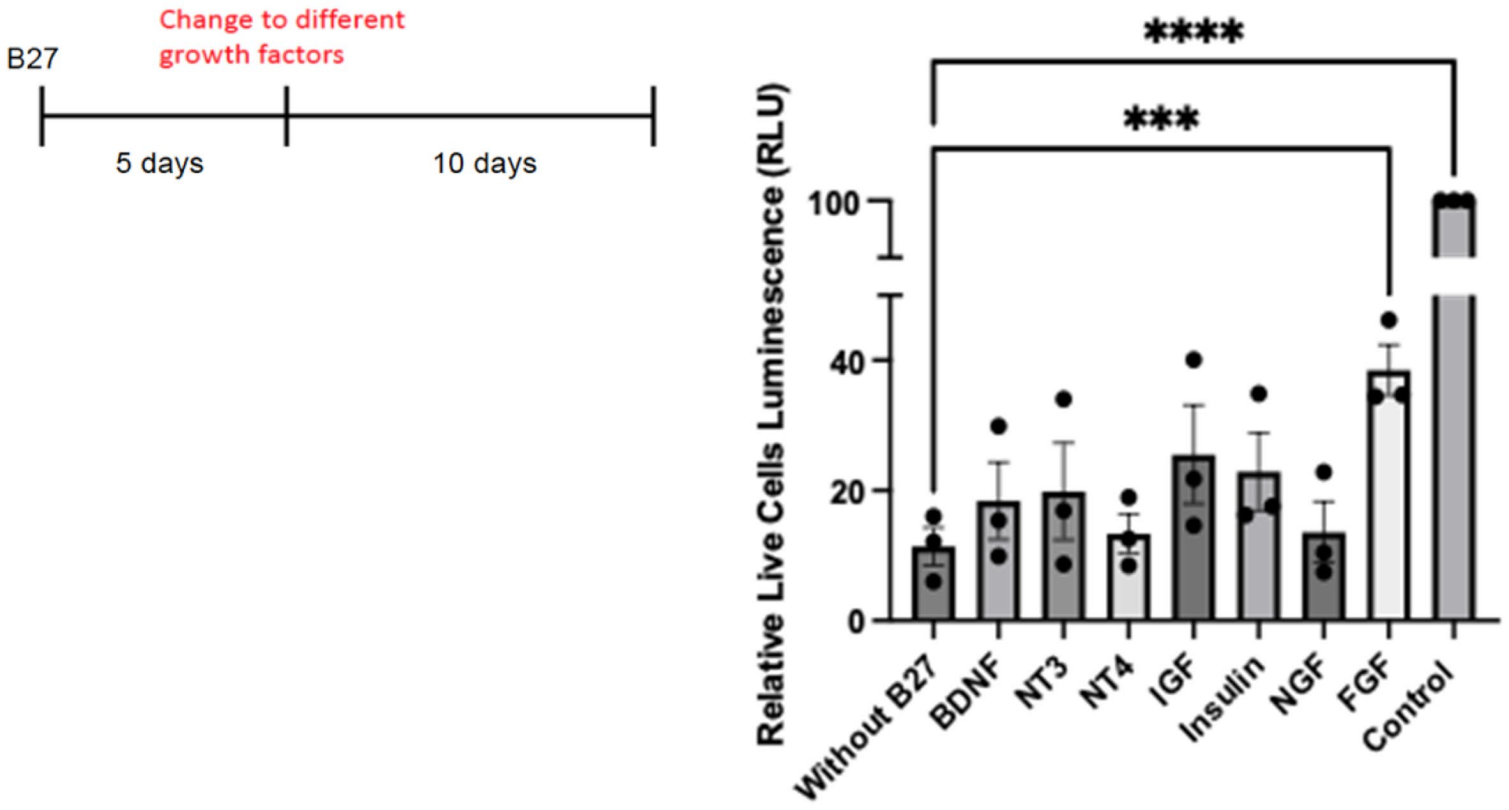
Hippocampal cells were plated on 48 well plates at 25,000/well. On day 5, complete B27 media was replaced with Neurobasal media lacking B27 with different neurotrophic factors and growth factors. For control cells, media was replaced with Neurobasal with B27 complete media. A cell survival assay was done after 10 days using the Promega CellTiter-Glo Luminescent Cell Viability Assay. Quantitation was carried out with Prism with one-way ANOVA. Growth factor concentrations used: BDNF 50 ng/mL, NT3 50 ng/mL, NT4 50 ng/mL, IGF 200 ng/mL, Insulin 4 ng/mL, NGF 50 ng/mL, and FGF 100 ng/mL. *** *p* value 0.0003 and **** *p* value < 0.0001.

To investigate whether individual growth factors might have an effect upon hippocampal neurons in culture, we tested whether any factors could rescue primary neurons in the absence of B27. To prolong the deprivation of B27, we extended the time of withdrawal to 10 days. Different known growth factors, such as IGF, insulin, FGF, and neurotrophins such as NGF, BDNF, NT-3, and NT-4 were tested for cell viability under this condition. Control cultures grown with B27 were monitored. With the exception of FGF, all the factors displayed statistically non-significant effects on cell survival (Figure 8). We have used deprivation of hippocampal neurons from lack of B27 (Lee and Chao, 2001; Jeanneteau et al., 2008) with reproducible results. However, despite much work on the viability of hippocampal neurons, a *bona fide* hippocampal growth factor has yet to be confirmed *in vivo*.

We are aware that B27 media is an artificial means of removing trophic support, as it contains multiple factors that change gene expression. Also, the heterogeneity and differential location of hippocampal neurons (dorsal-ventral, proximal distal) adds to a considerable diversity of cells in the subfields of the hippocampus (Cembrowski and Spruston, 2019). To assess whether the B27 treatment is capable of giving interpretable results, a bulk RNA-seq analysis of cells treated and untreated with B27 media was carried out.

**Figure 9:**
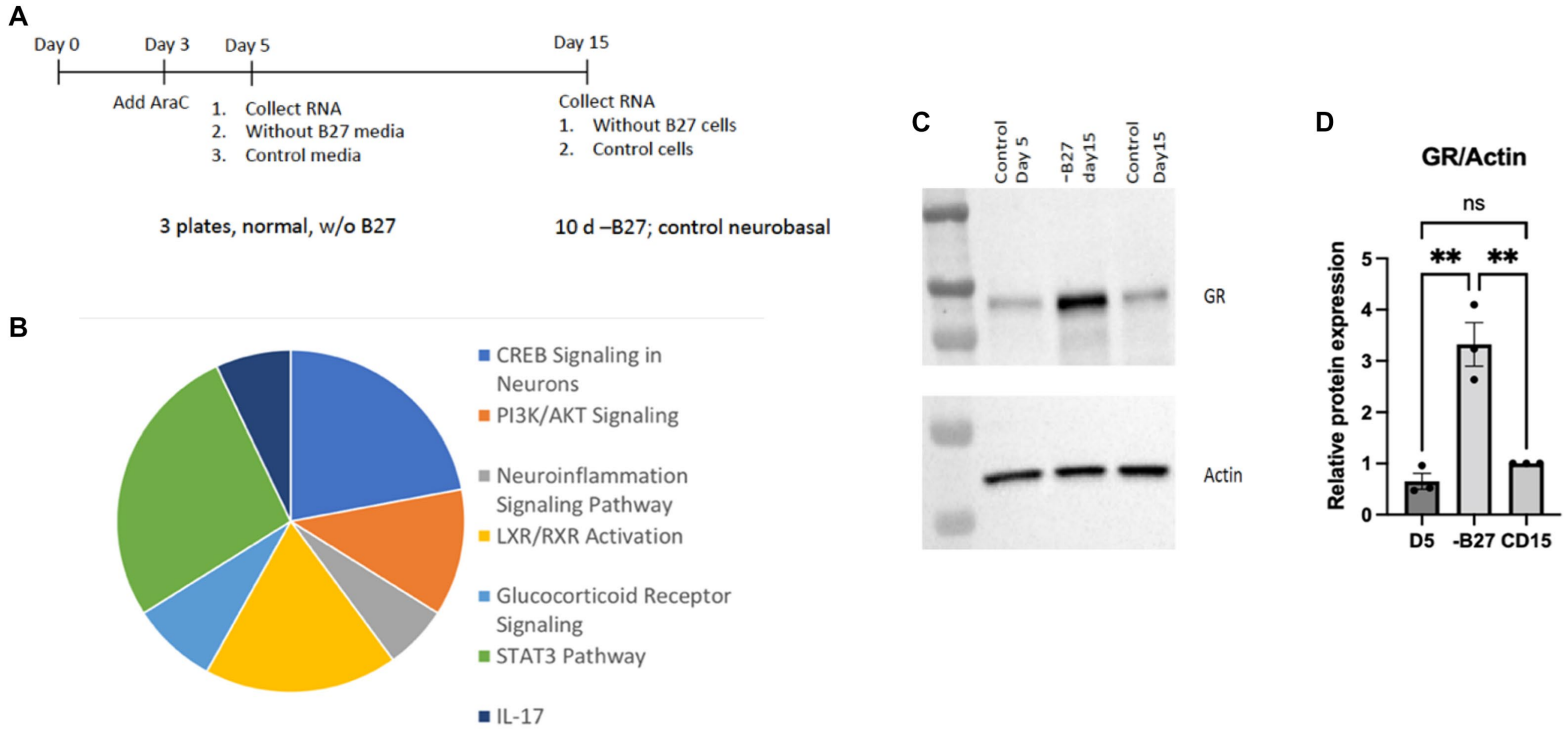
RNA-seq analysis of hippocampal neurons after deprivation of B27 media yielded signaling pathways of potential relevance. **(A)** On day 5, B27 media was withdrawn for 10 days from one set of neurons and then RNA was isolated. RNA was also collected from neurons after 5 days as a control. Another control was kept with B27 for additional 10 days (15 days total) before RNA was isolated. The RNAseq experiment was carried out with these three samples. **(B)** The pie chart lists pathways whose components were increased in hippocampal neurons in the absence of B27 media. **(C)** The absence of B27 (−B27) enhanced the expression of the glucocorticoid receptor (GR). Additional western blots are in Supplementary Figure 2. **(D)** Quantitation of GR levels in is shown in hippocampal neurons, D5 day 5; CD15, control day 15. The quantitation was carried out with Prism using one-way ANOVA. D5 vs. -B27 ** *p* value 0.0022; -B27 vs. CD15 ** *p* value 0.0037.

Several molecules were found to be increased in the absence of B27 media that reflected key regulatory activities, such as CREB, PI3K/AKT, STAT3, and IL17-related transcripts. Pathways associated with inflammation and steroid receptors—were also detected in the pie chart presented in Figure 9. Another signaling pathway with an appreciable increase is the glucocorticoid receptor (GR) that was surprisingly enhanced in the absence of trophic support by B27 (Figures 9B,C). The glucocorticoid pathway represents a major stress response, which may represent a reaction to the lack of trophic support.

### Glucocorticoid receptor, a responsive gene

The increase in GR upon withdrawal of trophic support in hippocampal neurons is a significant finding. We verified the GR protein was highly induced in hippocampal neurons after removal of trophic B27 trophic support (Figure 9C). The hippocampus is a main target of corticosterone and several populations of hippocampal neurons abundantly express GR. Glucocorticoids (GCs) produce both protective and toxic effects in the nervous system. In excess, GCs produce neuronal damage after stress; however, the neuroprotective effects of adrenal steroids also have been reported. Neuroprotective effects of glucocorticoids can be observed throughout the brain (Abraham et al., 2001).

Deprivation of nutrients from cultured hippocampal neurons after removal of B27 trophic support gave a significant induction of the GR protein in hippocampal neurons. Further documentation of protein expression of GR is shown in Supplementary Figure 2. Several potential mechanisms have been proposed for why there is an increase in GR from the lack of trophic support. One explanation is to provide signals for neuroprotection. Glucocorticoids are capable of transactivating the TrkB receptor for BDNF and promoted neuronal cell survival via a mechanism dependent on GR transcriptional activity. We have found that glucocorticoids can activate TrkB neuroprotective signaling independent of neurotrophins (Jeanneteau et al., 2008). Moreover, BDNF and glucocorticoid cooperatively regulate gene expression in the hippocampus. Transcriptome analysis indicated that a majority of glucocorticoid-responsive genes overlapped with BDNF-regulated genes in the CNS (Lambert et al., 2013). Although GCs are potent enhancers of apoptosis and can potentiate glutamate excitotoxicity in hippocampal neurons, GCs also provide a protective effect in the absence of trophic support. This would represent an innovative way for an increase in GR to function in the hippocampus in the absence of BDNF and trophic support.

## Discussion

To survive in a changing environment, all cells and organisms must be able to adapt both metabolic and gene expression programs. Accomplishing these goals requires sophisticated sensing mechanisms to detect fluctuations from a wide range of nutrients and energy supplies, necessary growth and survival factors required for trophic support. Responsiveness is transmitted through remodeling signal transduction pathways and alterations in gene expression. We speculate that changes in neuronal responsiveness during development and aging will give insight during the absence and presence of trophic support.

Deprivation-induced death of sympathetic neurons is also dependent upon mitochondrial functions that control neurotransmitter release, neurite outgrowth and dendritic remodeling, in addition to apoptosis (Chang et al., 2002). Recent evidence suggests that NGF exerts neurotrophic activity by influencing cell homeostasis and metabolism (Sun et al., 2017; Colardo et al., 2022). As mitochondria are ATP generating organelles that are distributed throughout the length of axons and in presynaptic terminals, they participate in fission and fusion events as well as neurite outgrowth (Cheng et al., 2010).

As the cells mature *in vitro*, neurons can develop a resistance to trophic factor deprivation and become much less dependent upon trophic factors. One caveat with studies of neurons placed in culture is that changes may occur over time that are no longer faithful replicas of their *in vivo* counterparts. The culture medium may not completely replicate the *in vivo* environment. For sympathetic neurons, the phenomenon of young to surviving mature cells after NGF withdrawal has been recapitulated *in vivo* (Angeletti et al., 1971; Goedert et al., 1978; Otten et al., 1979).

It is also plausible that other cell types are associated with sympathetic ganglia which may exert an effect on sympathetic neurons through secreted cytokines and growth factors, such as TNF, FGF4, TGFβ, and IGF-1 (described in Figure 3B). In particular, the cells include recently described sympathetic satellite glia (Mapps et al., 2022). This recent study did not evaluate the consequences of NGF withdrawal by the satellite glia and awaits further research.

For hippocampal neurons, removing the B27 media is an artificial means of providing trophic support. As B27 contains many factors that can change gene expression, it is an unnatural way of removing trophic support. We have used the lack of B27 nutrients previously (Lee and Chao, 2001; Jeanneteau et al., 2008) as a means to study the consequences of deprivation of hippocampal neurons. To align and verify the cell culture approach, microarray experiments to compare with the glucocorticoid transcriptomes have been undertaken (Lambert et al., 2013; Mariga et al., 2014). These experiments confirmed and aligned the glucocorticoid transcriptome with the genomic effects of BDNF (Lambert et al., 2013).

The requirements of sympathetic neurons in the PNS are critically dependent upon NGF for survival. In contrast, hippocampal neurons in the CNS are heterogenous and display differential vulnerability to neurodegeneration depending upon their location in different subfields (Roussarie et al., 2020). A fundamental difference between PNS and CNS neurons is the complexity of their afferent and efferent connections. In contrast to peripheral neurons, the CNS neurons are connected to afferent axons from many cell types. Survival of CNS neurons is usually not dependent upon a single trophic factor. The switch in gene expression from trophic a lack of support of these neuronal populations is not an artifactual event. An increased resistance to trophic factor loss has been described for several neuronal populations following injury, axotomy, or following infection.

A number of different neuronal populations display an increased resistance to cell death or injury. Motor neurons subjected to nerve crush in perinatal animals results in cell death, however, older motor neurons become less vulnerable (Kuzis et al., 1999). As another example, transection of motor neurons leads to death during early postnatal periods, but the majority of motor neurons can survive for prolonged periods (Snider and Thanedar, 1989). Likewise, retinal ganglion cells undergo rapid cell death postnatally after optic nerve transection whereas adult cells are more resistant (McKernan et al., 2006). The ability of neurons to develop resistance to apoptosis may be related their state of maturity and the type of injury.

To model cell type-specific vulnerability, it is important to examine differences between healthy neurons and those that become resistant to deprivation or toxicity. The inability of mature neurons to maintain or lose resistance to trophic factor deprivation may underlie the development of neurodegenerative diseases and psychiatric disorders (Autry and Monteggia, 2012; Allen et al., 2013). Trophic factor deprivation has been frequently associated with neurodegenerative diseases and apoptosis. Through many profiles have been conducted with genes representing Aβ and tau, the molecular basis for selective vulnerability remains unknown. In addition to the neuronal survival function of Trk receptors, the p75 receptor can exert a neuroprotective role *in vivo* on the Aβ-amyloid-damaging effects in sympathetic neurons (Bengoechea et al., 2009).

Previously we used deprivation of hippocampal neurons from lack of B27 with consistent results to identify small molecule ligands of GPCRs that provide a survival outcome (Lee and Chao, 2001; Jeanneteau et al., 2008). The interest in the glucocorticoid receptor (GR) is through its role as a ligand-activated transcription factor that mediates many physiological processes, including stress and inflammation. In the dentate gyrus (DG) of the hippocampus, both TrkB and GR proteins are expressed by granular cells. TrkB is found at high levels in dendrites and axonal projections of the hilus, and GR is found in the soma and nucleus.

Glucocorticoids (GCs) like corticosterone display both protective and adverse effects in the nervous system. The reasons for the dichotomy of these effects are not fully understood. In excess, GCs produce neuronal damage after stress or injury; however, neuroprotective effects of adrenal steroids have been reported (Sloviter et al., 1989; Abraham et al., 2001). Our previous studies showed that GR is a potent target of dexamethasone and corticosterone in the survival of hippocampal and cortical neurons (Jeanneteau et al., 2008). The hippocampus is a main target of corticosterone. These findings provided evidence that withdrawal of trophic support provides a mechanism for changes in gene expression through a known GR transcription factor with genomic and non-genomic effects (Panettieri et al., 2018).

Stress and release of glucocorticoids are common responses to injury and disease, and can represent modifiers of synaptic plasticity (Sorrells and Sapolsky, 2007). Indeed, several studies have supported positive effects of stress through the glucocorticoid receptor on axon growth and regeneration (Madalena and Lerch, 2016; Lerch et al., 2017). We suggest the deprivation of nutrients we used in this study is a form of stress on hippocampal neurons.

We are intrigued by the possibility that GR might likewise serve in a neuroprotective role for hippocampal neurons. We previously used deprivation of hippocampal neurons from a lack of B27 with consistent results to identify small molecule ligands of GPCRs that provide a survival outcome through transactivation of TrkB receptors (Lee and Chao, 2001; Jeanneteau et al., 2008). In the dentate gyrus (DG) of the hippocampus, both TrkB and GR proteins are expressed by granular cells. TrkB is found at high levels in dendrites and axonal projections of the hilus, and GR is found in the soma and nucleus (Lambert et al., 2013).

Deprivation of hippocampal neurons has revealed other genes. Previous hippocampal primary culture studies after withdrawal of BDNF identified regulated genes from a microarray analysis. Another protein, Narp/Nptx, was found in hippocampal neurons deprived of BDNF (Mariga et al., 2014). Narp/Nptx is downregulated directly by loss of BDNF and is responsible for the generation of mossy fiber LTP in the dentate gyrus (Mariga et al., 2015). In addition, Narp/Nptx levels are lower in CSF and tissues associated with Alzheimer’s disease (Hanson, 2017; Xiao et al., 2017). Hence, identification of hippocampal genes from a lack of trophic support sheds light into potential mechanisms of neurodegeneration.

The switch in dependence upon trophic support has been relevant therapeutically in clinical trials on pain management in human subjects. A notable example for the deprivation of trophic factors *in vivo* comes from anti-NGF antibodies to block pain. Clinical trials with tanezumab, a monoclonal blocking antibody against NGF, demonstrated that hyperalgesia was effectively reduced (Lane et al., 2010). During inflammation and tissue damage, NGF levels are increased in many peripheral tissues. This leads to a peripheral hypersensitization of nociceptive sensory neurons, leading to long lasting pain sensitivity in humans (Denk et al., 2017). Blocking NGF activity via administration of anti-NGF antibodies decreased pain-related behavior in animal models (Mantyh et al., 2011).

Current attempts to use anti-NGF antibodies and inhibitors have been successful in clinical trials of osteoarthritis, low back pain, and cancer-related pain. Antibodies that sequester NGF have been effective for the treatment of chronic pain conditions (Lane et al., 2010; Barker et al., 2020). This approach was based upon the ability of NGF to mediate persistent pain states by sensitizing nociceptive sensory neurons (McMahon, 1996).

Several concerns have been raised about potential toxic effects of anti-NGF (Lane et al., 2010; Wood, 2010). Indeed, one question is whether the use of antibodies to deprive NGF will lead to cell death as in the case of embryonic sympathetic neurons. However, administration of tanezumab did not result in cell death of sympathetic neurons in adult monkeys. Blocking NGF did not lead to adult sympathetic neuronal cell death (Belanger et al., 2017), consistent with the primary culture results presented here.

Administration of anti-NGF antibodies block the hypersensitivity to pain from excess NGF produced after injury and inflammation. The presence of viable sympathetic neurons with anti-NGF treatment is consistent with survival mechanisms in mature neurons after withdrawal of NGF. Why sympathetic neurons do not need NGF for trophic support in adult states, whereas NGF inhibition during development leads to sensory and sympathetic neuronal cell death is a question that has not been completely answered.

In the adult state, NGF is no longer required for survival of sensory or sympathetic neurons. We have shown that sympathetic neurons are capable of being rescued from NGF withdrawal through increased expression of genes in the mature state. It is significant that blocking NGF in the adult did not lead to sympathetic neuronal cell death or changes in sympathetic function (Belanger et al., 2017).

Future studies of the transcriptional and proteomic changes in sympathetic and hippocampal cultures deprived of trophic support will provide an indication of the parameters that lead to independence from trophic factors. A surprising outcome is that although growth factors keep neurons alive, the cells become less and less dependent on these factors to function over time. Our results demonstrate the feasibility of uncovering elements to explain the durability of neurons. As mature neurons develop a resistance to trophic factor deprivation, these experimental results suggest mechanisms that account for longevity may potentially explain how neurons can be active across a lifespan.

## Materials and methods

### Animals and primary neuron cultures

All experiments are conducted in accordance with the guidelines of the NIH (DHHS Guide for the Care and Use of Laboratory Animals: 1985) and the Guidelines for the Use of Animals in Neuroscience Research by the Society for Neuroscience. The animal research experiments were approved according to the NYU Institutional Animal Care and Use Committee (IACUC) of New York University Langone Medical Center. Primary cortical neurons were isolated from E18 rat embryos, cultured on poly-D-Lysine coated coverslips or 6-well plates, and maintained for a week *in vitro* in Neurobasal medium containing B27 supplement, 0.5 mM L-glutamine.

### Primary sympathetic neurons

Methods of culturing rat embryonic sympathetic neurons for short times and 3–4 weeks are followed from established procedures (Easton et al., 1997). For NGF deprivation, young neurons are first cultured in Neurobasal Media (NBM medium) for 5 days, then NGF is removed NGF by rinsing once with NBM medium followed by replacing the medium. Control neurons are treated similarly; their medium was replaced with NBM medium plus 50 ng/mL 2.5S NGF. For mature sympathetic cultures, cells were cultured in NBM medium plus 50 ng/mL 2.5S NGF for 14–28 days, then NGF was removed. In an initial profile for the sympathetic transcriptome, we initiated bulk RNA-seq experiments on sympathetic neurons that are grown under young and longer culture conditions (Figure 1). In preliminary results, we will focus on sympathetic cultures grown for 5 days, followed by removal of NGF for 6 h (immature, young).

### Quantitative assay for cell viability of sympathetic neurons

Superior cervical ganglia neurons are isolated from rat E21 pups. Neurons are grown in 96 well plates with NGF for 5 days then changed to either NGF, BDNF, GDNF, or without growth factors for 7 days. Cell toxicity assay is performed by using CytoTox-Glo Cytotoxicity Assay kit (Promega, G9291). CytoTox-Glo™ Cytotoxicity Assay Reagent (50 μL) is added for 15 min at room temperature and then luminescence is measured (experimental cell death luminescence). The activity of a dead cell protease activity is measured in degenerating cells. After treatment, 50 μL of Lysis Reagent is added and the luminescence (total luminescence). The luminescence for viable cells is calculated by subtracting the luminescent signal from experimental cell death from total luminescence death.

### Cultured hippocampal neurons

Dissociated primary cultures of hippocampal neurons from embryonic day 18 (E18) are prepared from timed-pregnant Sprague–Dawley rats. All dissection work is carried out in ice-cold buffer (1X PBS, 10 mm HEPES, and 0.6% glucose, pH 7.35). Cells are plated at a density of 1,000,000 per 6-cm dish in plating medium. Cells are treated with Ara C after 3 days in culture. B27 withdrawal procedure (Figure 7). At day 5, the complete (Neurobasal +B27) media is replaced with only Neurobasal media. The cells are allowed to be mature for next 10 days. At day 15, one set of cells are collected and a protein lysate prepared for western blotting, and second set of cells are used for RNA extraction followed by RNAseq analysis. Primary neurons were cultured on poly-D-lysine, maintained in Neurobasal media containing B27 supplement, 0.5 mM L-glutamine, 5-fluoro-uridine, and uridine (10 mM each). During starvation from B27, 1 μM MK-801 was added to decrease the contribution of *N*-methyl-D-aspartate-mediated cell death.

### Antibodies for immunostaining

Antibodies against NRH2 were generated by the laboratory of Chao (2003) and previously published (Murray et al., 2004). An antibody against the Glucocorticoid Receptor (D8H2) XP^®^ Rabbit mAb #3660 was from Cell Signaling. Primary neurons were fixed on coverslip and incubated with primary antibodies in donkey serum/PBS/Triton at 4°C overnight, followed by incubation with secondary antibodies.

### Statistical analysis

The results represent the average of at least three independent experiments, unless indicated. Statistical significance was determined by One-way repeated measures ANOVA or Student’s test. All statistical analyses of quantitative data are expressed as mean ± SEM. The expression of oxytocin receptors was measured by the use of receptor antibodies generated in our lab. Trk receptors were measured by anti-phosphotyrosine antibodies. Quantification of immunolabeled sections is performed in a blinded fashion on images with statistical analysis. For each protein measurement, the statistical significance is measured. The *p* values equal to or less than 0.05 were considered significant, asterisks denote statistical significance (^*^p < 0.05; ^**^p < 0.01; ^***^p < 0.001). *p* values are calculated using either two-tailed unpaired Student’s *t*-test or ANOVA.

### RNA seq experiments

We initiated bulk RNA-seq experiments on sympathetic and hippocampal neurons that are grown under young and mature culture conditions (Figures 1, 8). The SCG is harvested from rat pups of two pregnant rats, dissociated and 100,000 cells plated in 12-well plate. Hippocampal cultures were made from E18 embryos, dissociated and 500.000 cells plated on six well plates. Cells are treated with antimitotic agents for 5 days and total RNA is harvested using Trizol. RNA extractions were quantified using RNA Nano Chips (Cat. #5067–1511) on an Agilent 2100 BioAnalyzer. RNA-Seq library preps were constructed using the Illumina TruSeq Stranded mRNA Library Prep kit (Cat #20020595) using 500 ng of total RNA as input, amplified by 11 cycles of PCR. Final libraries were visualized using High Sensitivity DNA ScreenTape (Agilent, Cat. #5067–5584) on the Agilent TapeStation 2200 instrument. Quant-It (Invitrogen, Cat. P11495) was used for final concentration determination and libraries were pooled equimolar. The pool was sequenced paired-end 50 cycles on an Illumina NovaSeq6000 SP 100 Cycle flowcell-v1.5 with 2% PhiX spike-in.

### Lentivirus

The envelope plasmid (pMD2.G), packaging plasmid (psPAX2), and control vectors were co-transfected into 293LTV cells and viral particles were harvested 48 h post-transfection. Lentiviral particles were added to sympathetic neurons overnight. After 7 days, cell lysates were collected for western blot.

## Data availability statement

The original contributions presented in the study are publicly available. This data can be found here: https://www.ncbi.nlm.nih.gov/bioproject/PRJNA949235.

## Ethics statement

The animal study was reviewed and approved by NYU School of Medicine IACUC Committee.

## Author contributions

H-LH and LK initiated the experiments, with the help of EC, MS, and CM. MC conceived of the project, supervised the experiments, and wrote the manuscript with the help of H-LH and LK. TH was a mentor for the project. All authors contributed to the article and approved the submitted version.

## Funding

The study was funding by NIH (grants R35GM139610 (TH), R01 MH119136, U19 NS107616, and R25NS107178) and the Simons Foundation (Project 328183).

## Conflict of interest

The authors declare that the research was conducted in the absence of any commercial or financial relationships that could be construed as a potential conflict of interest.

## Publisher’s note

All claims expressed in this article are solely those of the authors and do not necessarily represent those of their affiliated organizations, or those of the publisher, the editors and the reviewers. Any product that may be evaluated in this article, or claim that may be made by its manufacturer, is not guaranteed or endorsed by the publisher.
