# Supplementary material for: Confronting the loss of trophic support
Source: Front Mol Neurosci. 2023 Jun 23;16:1179209. doi: 10.3389/fnmol.2023.1179209 (PMC10338843; doi:10.3389/fnmol.2023.1179209)
Supplement: Supplementary file 1 [file Data_Sheet_1.docx]

**Supplementary Data**

**Figure 1**


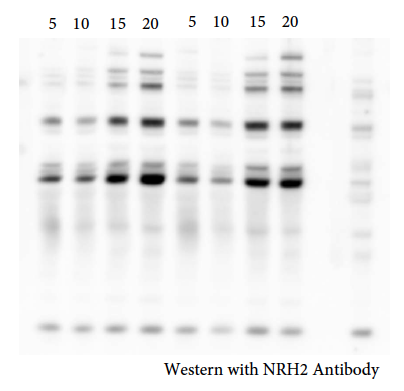


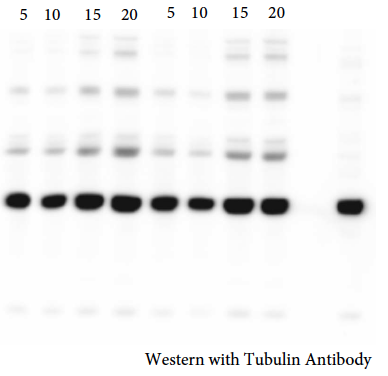


**Figure 1**. Western blot of lysates from mature sympathetic samples after 5 to 20 days in culture were analyzed with antibodies against NRH2 (left) and tubulin (right). Two separate time course experimental lysates were analyzed after loading on SDS-PAGE gels and transfer to NC membrane. The arrow designates the NRH2 protein.

**Figure 2**


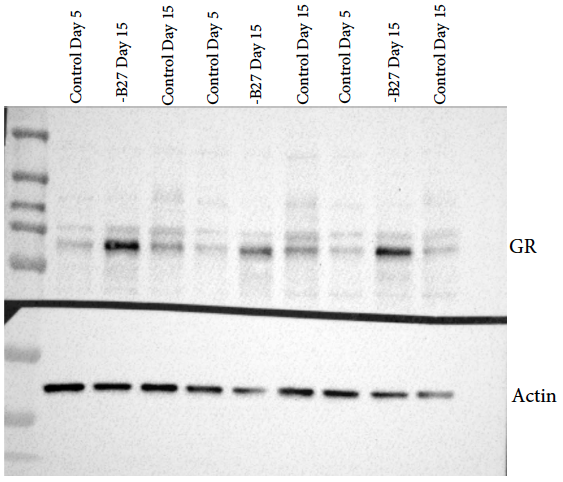


**Figure 2.** Western blot of 3 separate experimental lysates from hippocampal cultures. Control lysates from primary neurons grown for 5 days; or 15 days without B27 supplement; or control hippocampal cells grown for 15 days. The samples were subjected on SDS-PAGE gels, transferred to nitrocellulose membranes and analyzed with antibodies against the Glucocorticoid Receptor (GR) and Actin.
